# Study on Permeability Stability of Sand-Based Microporous Ceramic Filter Membrane

**DOI:** 10.3390/ma12132161

**Published:** 2019-07-05

**Authors:** Wei Zhou, Lin Zhang, Pute Wu, Yaohui Cai, Xiao Zhao, Chunping Yao

**Affiliations:** 1 Key Laboratory of Agricultural Soil and Water Engineering in Arid and Semiarid Areas, Ministry of Education, Northwest A&F University, Xianyang 712100, China; 2 Institute of Soil and Water Conservation, Northwest A&F University, Xianyang 712100, China; 3 Institute of Water Saving Agriculture in Arid Areas of China, Northwest A&F University, Xianyang 712100, China

**Keywords:** microporous ceramic, flow rate, filter, membrane, permeability stability

## Abstract

The instability of diafiltration is a widespread problem in the practical application of microporous ceramic filtration membranes. In this paper, a series of microporous ceramic filter membranes were prepared using inexpensive standard sand and river sand as matrix materials. Semi-empirical formula for the effective permeability radius of ceramic membranes with respect to time was established from analysis of the response mechanism between water flow and material properties. Finally, on the basis of theoretical analysis, some measures were proposed to improve permeate flux. The experimental results showed that during the initial stage of filtration, the microporous ceramic filter membrane had a large change in permeate flux, and during the late stage of filtration, permeate flux tended to be stable. Over time, open porosity and closed porosity changed the actual seepage area of the ceramic membrane, and this affected the stability of permeate flux and final stable permeate flux. The roughness of the inner wall of microporous ceramic pores affected the hydraulic loss coefficient, and this controlled the outflow process. Trace elements that were rich in sand produced a large amount of glass phase after sintering. The glass phase was rich in polar groups and formed a temporary hydrogen bond with the small flow of water molecules. It led to an increase in viscous resistance effect of the side wall along the water flow and the extent of the permeate flux of the ceramic membrane changed with time.

## 1. Introduction

Membrane filtration is an effective method for removing particulates, microorganisms, and organic matter from wastewater [1]. Membrane treatment (i) provides higher water quality, (ii) minimizes disinfectant requirements, (iii) is more compact, (iv) provides easier operational control and less maintenance than conventional treatments, and (v) produces less sludge [2,3,4,5,6]. Conventional microfiltration membranes are mostly prepared using polymers, but they are costly and have poor durability [7]. In recent years, microporous ceramics have been widely used in the preparation of microfiltration or ultrafiltration materials because of they have the advantages of heat resistance, chemical resistance, mechanical resistance, controllable microstructure, and low environmental pollution [8,9,10].

At present, researchers at home and abroad have done a lot of studies using ceramic membranes for seepage [11,12,13]. Sui used a centrifugal casting method to prepare a gradient diatomaceous earth film that can remove 100% of pathogenic bacteria, rust bacteria, worms, and suspended particles in water; it can also recover the partial flux efficiency of a ceramic membrane via simple mechanical brushing [14]. Jang used a support layer of alumina to prepare a microporous ceramic membrane that effectively filters particles and exhibits acceptable water permeability [15]. Although Jang’s ceramic membrane exhibits a high influent recovery rate and a low chemical cleaning frequency in seepage, when the ceramic membrane was used for seepage, the permeate flux stability is poor in practical applications. According to Lee’s research, the permeate flux of the porous ceramic membrane decreased by about 90% in the first 2 h of seepage [16]. Cui used a laboratory-scale ceramic membrane device to study the performance of ceramic membranes that had different pore sizes and reported that after 40 h, the permeate flux is only 15% of the initial value [17]. Although Lee and Cui used clear water with a low impurity content as the over-flow medium, the penetration flux fluctuates greatly with time, and this phenomenon reduces the permeate flux of the membrane as well as the efficiency of sewage treatment.

To improve the permeate flux stability of the ceramic membrane, Li sprayed a nano-zinc oxide suspension which was an adjustable adhesive onto the desired substrate and successfully prepared a super-hydrophobic zinc oxide surface [18]. Cagla dispersed the hydrophobic fumed silica in an organic solvent and continuously spin-coats the polymer film to prepare a super-hydrophobic polymer surface [19]. In addition, methods such as electrospinning [20], chemical vapor deposition [21], chemical etching [22], and electrochemical deposition [23] can be used to combine micron or nano-level structures with low-surface energy materials to prepare super-hydrophobic surfaces. Although these methods effectively improve the permeation performance of the ceramic membrane, they are expensive, and the preparation processes are relatively complicated. Thus, such methods are rarely used on a large scale for industrial and civilian use. However, there are few studies that propose approaches to improve the stability of the permeate flux of the microporous ceramic filter membrane, with a theoretical perspective.

Therefore, we used river sand and standard sand as matrix materials and analyzed the water flow and response mechanism of the ceramic membrane substrate materials to establish a preliminary variation law of the effective permeability radius of the ceramic membranes with respect to time. Based on this, we proposed some reasonable approaches to improve the permeability of ceramic membranes.

## 2. Experimental Procedure

### 2.1. Sample Preparation

**Figure 1:**
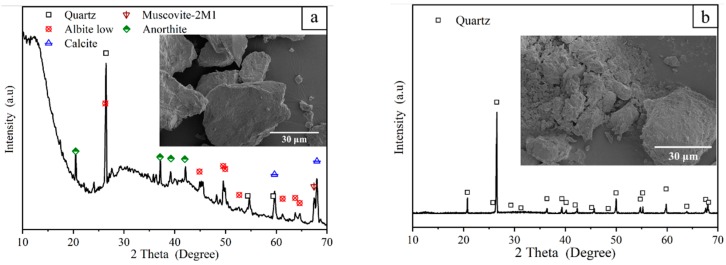
SEM (Scanning electron microscope) image and XRD (X-ray diffraction) pattern of (**a**) river sand and (**b**) standard sand.

**Figure 2:**
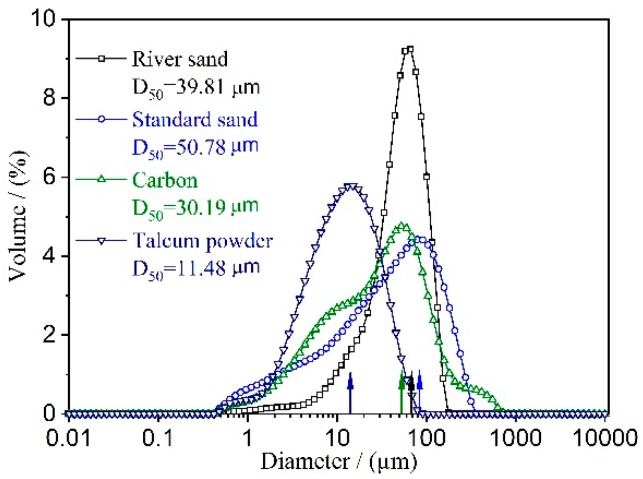
Particle size distribution of raw materials.

River sand (see Table 1 for the main components; third-order wetland from the Wei He River) and standard sand (see Table 1 for the main components; produced by Xiamen ISO Standard Sand Co., Ltd., Xiamen, China) were each used separately as the matrix material for the RS (River sand) series and the SS (Stand sand)series of samples, respectively. High-purity alumina powder (Al_2_O_3_, Tianjin Ko Mi Ou Chemical Reagent Co., Ltd., Tianjin, China), slag (see Table 1 for the main components; Institute of Water-Saving Agricultural Research in Arid Regions of China, Xianyang, China), talc powder (MgSiO_3_, Tianjin Zhi Yuan Chemical Reagent Co., Ltd., Tianjin, China), silica sol (AM30, SiO_2_ mass fraction of 30% ± 1%), and SiO_2_ (average particle size of 8–20 nm, Na_2_O mass fraction <0.05%, Shandong Bai Te New Materials Co., Ltd., Linyi, China) were used as additives. The prepared powder was added to a suitable silica sol according to the ratio shown in Table 2 and stirred to prepare a powder, which was then pressed in a circular tube (20 mm inner diameter and 40 mm outer diameter) at a pressure of 12 MPa (Figure 1a). The pressed green body was air-dried for several days in a cool and ventilated place and then placed in a high temperature box furnace (KSL-1400X-A3, Hefei Ke Jing Material Technology Co., Ltd., Hefei, China). After several cycles of heating at constant temperature, the tubular microporous ceramic filter membrane was obtained by cooling the sample to room temperature. The particle size distribution of the raw materials is shown in Figure 2.

### 2.2. Sample Characterizations

#### 2.2.1. Mechanical Performance Characterization

The porous ceramic samples were ground, polished, wetted, and dried; then the porous ceramic samples were coated by ion beam sputter deposition (MC1000, Hitachi, Tokyo, Japan) and the microstructures were observed using a scanning electron microscope (Nova Nano SEM-450, FEI, Brno, Czech). Five microporous ceramic samples were randomly selected from each treatment group, and these samples were ground into powder, mixed, and placed on an X-ray diffractometer (X‘Pert Pro, Philip, Amsterdam, the Netherlands; scanning angle of 10°–70° and scanning speed of 5°/min) for phase and crystal structure analysis (the Inorganic Crystal Structure Database). Chemical composition was determined by spectrometry X-ray fluorescence (XRF1800, Shimadzu, Kyoto, Japan). The bending strength was determined using a mechanical testing machine (CMT4204, Xin San Si, Shenzhen, China). The sample dimensions were 3 mm × 4 mm × 40 mm, the span was 30 mm, and the loading speed was 0.5 mm/min. The volume density (*ρ_b_*) and open porosity (*e_o_*) of the porous ceramic samples were measured using the Archimedes method and Equations (1) and (2). The test true density (*ρ_t_*) (according to ISO5018-1983 standard) and the total porosity (*e_t_*) were derived using Equation (3). The closed porosity (*e_c_*) was calculated using Equation (4), and the radial shrinkage rate (*l*) was calculated using Equation (5).

(1) $$\rho_{b}=m_{s}/\left(m_{w}-m_{f}\right)\;\times \;100\%$$

(2) $$e_{o}=\left(m_{w}-m_{s}\right)/\left(m_{w}-m_{f}\right)\;\times \;100\%$$

(3) $$e_{t}=\left(1-\rho_{b}/\rho_{t}\right)\;\times \;100\%$$

(4) $$e_{c}=e_{t}-e_{o}$$

(5) $$l=\left|l_{2}-l_{1}\right|/l_{1}\;\times \;100\%$$

where: *m_s_* is the mass of a sample under dry conditions (g), *m_f_* is the sample float capacity of a sample (g), *m_w_* is the saturate bulk density of a sample (g), *l*_2_ is the diameter of a sample after sintering (mm), and *l*_1_ is the diameter of a sample before sintering (mm).

#### 2.2.2. Hydraulic Performance Characterization

**Figure 3:**
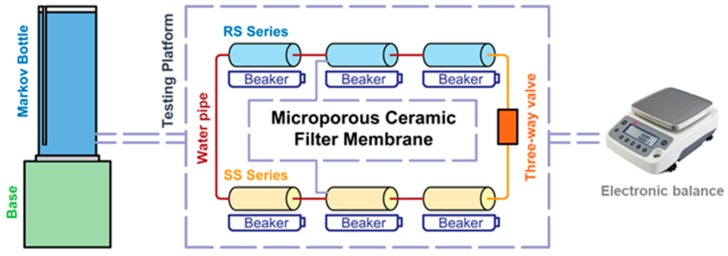
Hydraulic performance test device.

The test device is shown in Figure 3. The water supply unit used a Martens flask, and the test device consisted of a test platform and a Ying Heng Electronic Balance. The microporous ceramic filter membrane was mounted on a hydraulic test platform, and the height of the base of the Martens bottle was adjusted to allow the membrane to percolate under working pressures of 20 cm and 50 cm. Before recording data, the three-way valve was opened. To ensure the accuracy of the test data, the microporous ceramic filter membrane was fully flowed for 30 min to eliminate air from the pores as much as possible. The water used in the laboratory was domestic water that is used by residents in Yang Ling. The water temperature was 25 °C. During the testing period, the permeate flux was recorded every 3 h; the permeate flux was not recorded at night. Each test was repeated three times.

## 3. Results and Analysis

### 3.1. Permeate Flux Q Changes with Time t

**Figure 4:**
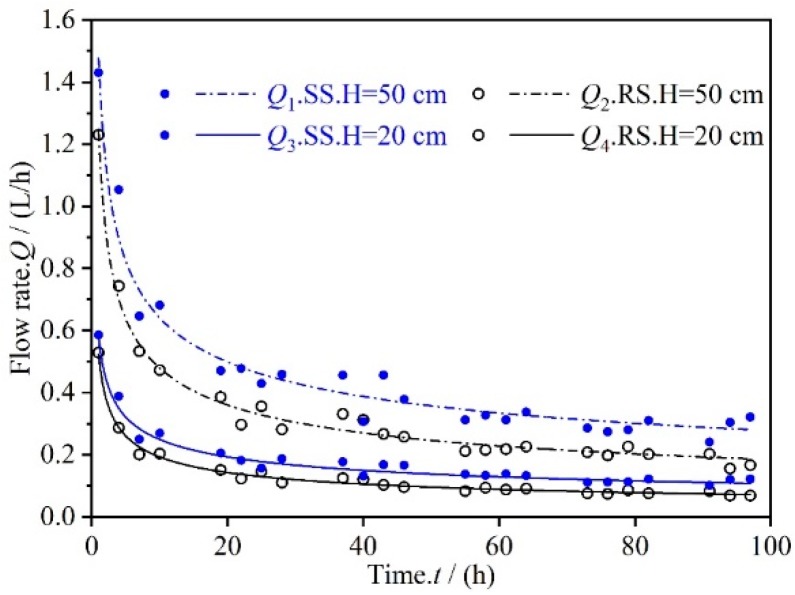
Changes in permeate flux with respect to time for RS and SS series of microporous ceramic filter membranes.

Figure 4 shows changes in the permeate flux of the RS and SS series microporous ceramic filter membranes with respect to seepage time at 1150 °C. Under different working pressures, the permeate fluxes of the RS and SS series samples had similar trends with respect to time; specifically both decreased unevenly over time. In microfiltration ceramics, the permeate flux of the filtration membrane changes greatly during the initial stage of filtration, and it tends to be stable during the later stage of filtration. Outflows of RS at working pressures of 50 cm and 20 cm are expressed as *Q*_1_ and *Q*_2_, respectively, and outflows of SS at working pressures of 50 cm and 20 cm are expressed as *Q*_3_ and *Q*_4_, respectively. In the first 40 h of seepage, the values of *Q*_1_, *Q*_2_, *Q*_3_ and *Q*_4_ decreased by 78.60%, 77.49%, 74.62%, and 77.10%, respectively. Between 40 h and 100 h of seepage, the values of *Q*_1_, *Q*_2_, *Q*_3_, and *Q*_4_ only decreased by 21.40%, 22.51%, 25.37%, and 22.90%, respectively. The working pressure has a certain influence on the flow rate of the microporous ceramic filter membrane. However, the degree of correlation between the two may not be very high, and thus, the influence of the working pressure on the permeability of the permeate flux is not discussed here.

Samples of the RS and SS series were sintered at 1150 °C as an example to study the hydraulic performance of the microporous ceramic emitter.

The flow–time relationship curves were fitted using the least squares method, and the obtained functional relationships were: *Q*_1_ = 1.48 *t*^−0.41^, *Q*_2_ = 1.24 *t*^−0.36^, *Q*_3_ = 0.58 *t*^−0.43^, and *Q*_4_ = 0.52 *t*^−0.38^. A function formula for the flow *Q*–time *t* relationship curves of the different series of microporous ceramic emitters is easily obtained:

(6) $$Q\left(t\right)=at^{b}$$

where *a* > 0 and *b* < 0. According to previous research results, the following suppositions can be made: the values of *a* and *b* are related to the material type, pore distribution characteristics, and trace element content of the microporous ceramic filter membrane itself. The effects of these factors on the change in permeate flux were analyzed.

### 3.2. Analysis of Factors that Affect Changes in Permeate Flux

#### 3.2.1. Porosity

**Figure 5:**
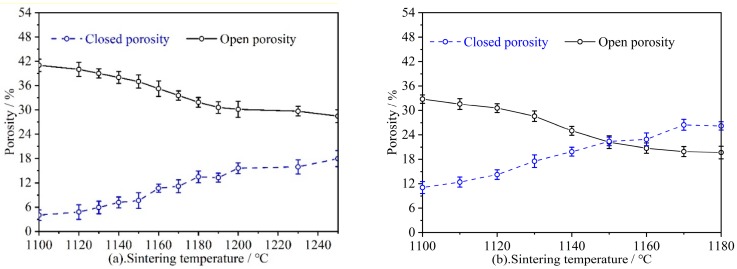
Porosity and flexural strength of (**a**) SS and (**b**) RS series samples at different sintering temperatures.

**Figure 6:**
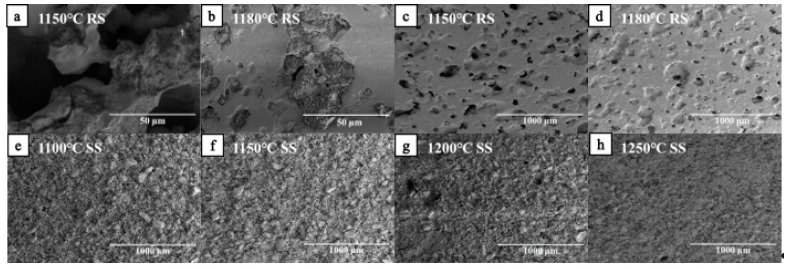
Microstructure diagram. (**a**) RS sintered at 1150 °C, 50 μm; (**b**) RS sintered at 1180 °C, 50 μm; (**c**) RS sintered at 1150 °C, 1000 μm; (**d**) RS sintered at 1180 °C, 1000 μm; (**e**) SS sintered at 1100 °C, 1000 μm; (**f**) SS sintered at 1150 °C, 1000 μm; (**g**) SS sintered at 1200 °C, 1000 μm; (**h**) SS sintered at 1250 °C, 1000 μm.

Figure 5 shows the relationship between the porosity of RS and SS series samples and the sintering temperature. The open porosity of the RS series samples slowly decreased below 1100 °C. When the sintering temperature was higher than 1150 °C, the sample melts (Figure 6a), and river sand transformed into a liquid phase that was rich in SiO_2_. The liquid phase filled in the gaps between the Al_2_O_3_ particles and resulted in a large number of closed cells. Thus, the open porosity was significantly reduced. When the sintering temperature was increased to 1180 °C and the sample was sintered for 2 h, the open porosity of the RS series was 19.67%, and the closed porosity was 26.20%. This indicates that most of the pores in the sample were closed pores. The open porosity of the SS series samples was similar to that of RS series. For the SS series samples, the open porosity decreased slowly from 1100 °C to 1120 °C; when the sintering temperature was between 1140 °C and 1200 °C, the open porosity rapidly decreased from 38.02% to 30.13%. When the temperature was above 1200 °C, the open porosity did not change much.

For the closed porosity in the RS series samples, when the temperature was increased from 1120 °C to 1150 °C, the liquid phase, unconnected pores, and closed porosity increased significantly; when the temperature was increased from 1150 °C to 1180 °C, the closed porosity is only increased slightly. With an increase in the sintering temperature, the compressive strength and closed porosity of the RS series samples increased. This indicates that an increase in the closed porosity had a greater influence on the compressive strength. Between 1100 °C and 1250 °C, the connection between the SS series of particles has been surface contact with no melting phenomenon. However, when the temperature was increased, the particle size of the quartz particles decreased, the edge broke, the closed porosity increased, and the particle contact was more complete (Figure 6e,h).

**Figure 7:**
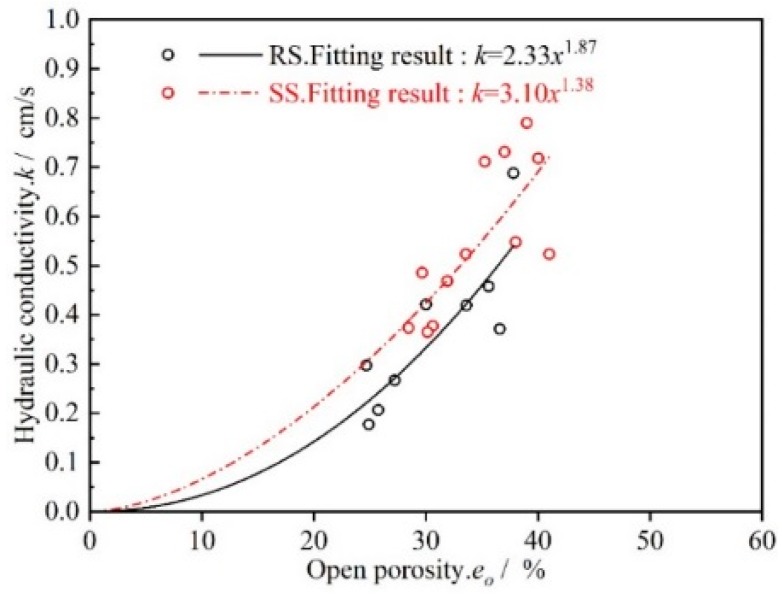
Relationship between the permeability coefficient (*k*) of the RS and SS series microporous ceramic filter membranes and the open porosity (*e_o_*).

In summary, when the temperature was increased, the open porosity (*e_o_*) gradually decreased, closed porosity (*e_c_*) gradually increased, actual outflow area of the tubular ceramic filter membrane decreased, and flow rate *Q* decreased. We can clearly conclude that open porosity and closed porosity affect the flow stability over time and that the amount of flow ultimately stabilizes permeation by changing the actual seepage area of the ceramic membrane. Therefore, it is necessary to plot the relationship between the permeability coefficient (*k*) and the open porosity (*e_o_)* to analyze the relationship between *k* and *e_o_* (Figure 7). From calculations, the permeability coefficient (*k*) of the microporous ceramic filter membrane and the open porosity (*e_o_*) satisfy a power function relationship, and this is consistent with the results reported by Cai et al. [24].

#### 3.2.2. Microstructure

As seen in Figure 6, after sintering at 1100 °C for 2 h, the SiO_2_ particles in the RS series sample were intact. Thus, densification of the ceramic structure is mainly achieved via structural rearrangement. At this point only a small portion of the pores were closed, and gaps formed a large number of open pores between the particles. When the sintering temperature was raised to 1120 °C, the RS particle boundary was still very obvious. Also, there was a large number of narrow holes, and this indicated that the RS particles mainly rely on bridging (Figure 6a). When the sintering temperature was increased to 1150 °C, the internal adhesion of SiO_2_ and Al_2_O_3_ particles in the microporous ceramic promoted the densification of the structure (Figure 6c). The number of narrow pores began to decrease, and the closed pores increased significantly, and the open pores significantly decreased. When the sintering temperature was increased to 1180 °C, the alkali metal oxide contained in the slag and river sand lowers the liquid phase formation temperature, and some of the river sand melts. The SiO_2_-rich glass phase is formed around the unmelted quartz particles and partially dissolved in the interstitial spaces between the Al_2_O_3_ particles (Figure 6b,d) [25]. Compared with 1150 °C (Figure 6c), the liquid phase that was rich in SiO_2_ inside the ceramic further diffused and filled with the gaps between the Al_2_O_3_ particles and the quartz particles, also, the distribution of the pores tended to be uniform. Therefore, at 1180 °C, the densification mechanism of the RS series was the transformation into a liquid phase encapsulation via simple bridging between particles. In contrast, the liquid glass phase formed by the melting of quartz has a lower viscosity, and this further improved densification of the structure of the interior of the microporous ceramic. At this point, SiO_2_ and Al_2_O_3_ were integrated, and there was no obvious bridging boundary observed. Molten SiO_2_ compensated for the position of some narrow pores, and this explains why the open pores decreased and the closed pores increased with an increase in temperature.

Figure 6f shows the microstructure of the SS series sample at 1150 °C. As seen from comparing Figure 6c,f, the SS series sample had a lower degree of densification than the RS series sample at the same temperature. In summary, there are two main reasons for this: First, SS had a larger particle size (D_50_ = 50.78 μm) than RS (D_50_ = 39.81 μm), and the larger particle size reduced the surface activity as the surface expanded. The increased diffusion distance slows the solid phase reaction rate of Al_2_O_3_ and SiO_2_ [26]. Second, impurities have a great influence on the densification rate and pore evolution of microporous ceramics. When the impurity content is high, more ions easily cover the surface of the quartz particles [27]. This results in an increase in the surface area of quartz, which inhibits the surface migration of the crystal and thereby reduces the densification rate of the powder and the growth rate of the crystal grains.

To some extent, the degree of fragmentation of the material particles determined the roughness of the pores inside the microporous ceramic. As seen in Figure 6, the surface of the RS series samples was significantly smoother than the SS series samples at the same sintering temperature. The difference in the roughness of the side wall causes a difference in the degree of the blockage of the water flow [28]. When the side wall is rough, the resistance coefficient λ inside the pore is larger, and the resistance along the path when water flows is also larger. The size of this influence changed over time.

#### 3.2.3. Phase Composition

**Figure 8:**
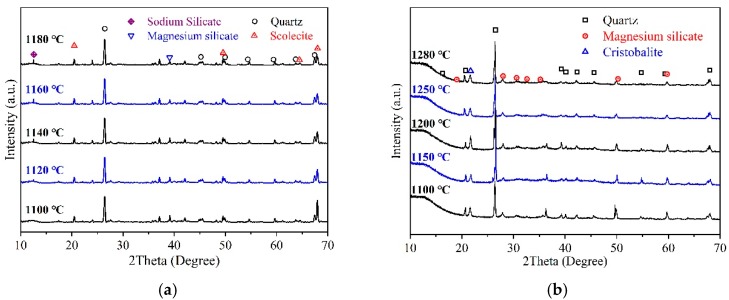
XRD patterns of (**a**) RS and (**b**) SS sintered samples at different temperatures.

The phase compositions of the RS series of samples that were sintered at 1100 °C to 1380 °C are shown in Figure 8a. The main crystal phase of the RS series was quartz (SiO_2_, hexagonal system, PDF#083-0542), and the secondary crystal phase was magnesium silicate (MgSiO_3_, orthogonal system, PDF#075-1406), calcium zeolite (Ca(Al_2_Si_3_O_10_)·3H_2_O, monoclinic system, PDF#085-0913), and sodium silicate (Na_2_SiO_3_, PDF#041-1335). When the temperature was increased, the composition of the composite silicate (magnesium silicate and sodium silicate) gradually increased, and therefore, the amount of the glass phase gradually increased. The phase compositions of the SS series after sintering the samples at temperatures from 1100 °C to 1280 °C are shown in Figure 8b. The main crystal phase of the SS series was quartz (SiO_2_, hexagonal system, PDF#083-0542), and the sub crystalline phase was magnesium silicate (MgSiO_3_, orthogonal system, PDF#075-1406) and cristobalite (SiO_2_, tetragonal system, PDF#082-0512). When the sintering temperature was increased from 1100 °C to 1250 °C, magnesium silicate gradually lost its structural water and became amorphous; thus, the content of magnesium silicate gradually decreased. Cristobalite is a phase change product that has poor thermal expansion properties, and it forms via α-quartz transformation above 573 °C [29]. The volume expansion rate caused by this process is as high as 15.4%. Thus, the volume expansion easily stress-damages inside the ceramic structure. An increase in temperature can increase the yield of cristobalite [30]; however, as seen from Figure 8b, when the sintering temperature was increased, the content of cristobalite did not significantly change. This may be because the amount of cristobalite produced was limited by the quartz content, and no different levels of quartz content were designed and assessed in this test.

Compared with the RS series samples, in the SS series samples, some of the quartz was converted into cristobalite at high temperatures. Therefore, at high temperatures, the amount of SS that converted to vitreous quartz was small, and the final glass phase content was low. This is also confirmed in Figure 6b,f. the glass phase and cristobalite have different degrees of affinity for polar molecules, and this is clear from the different viscous drag effects that the side walls have on the water flow. According to Newton’s internal friction law, *F = μ(du/dy)A*, when a material has a larger adsorption force on water, the viscosity coefficient *μ* is larger, and thus, the tangential force (*F*) that is generated between the side wall and the water flow is larger. When the tangential force is larger, the water flow of the ceramic membrane decreases. Therefore, the process of how the permeability coefficient (*k*) changes is related to the viscous drag effect of the side wall. In the sand-based microporous ceramic, the indirect coefficient (*k*) decreased when the glass phase content increased.

## 4. Approaches for Improving the Permeability of Ceramic Membranes

### 4.1. Theoretical Analysis of Permeate Flux Changes

**Figure 9:**
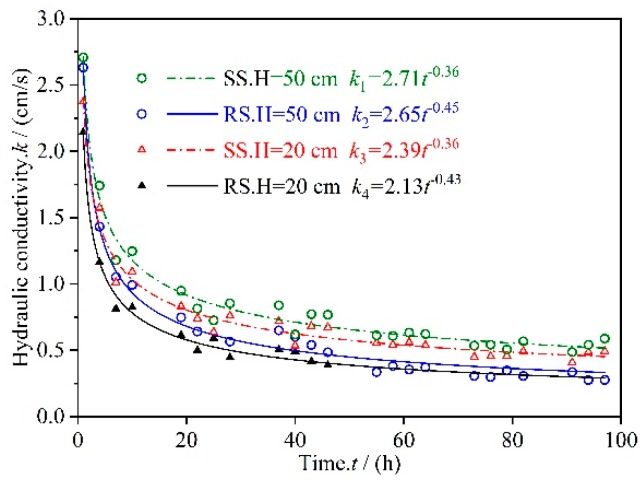
Changes in the permeability coefficient for RS and SS series of microporous ceramic filter membranes with respect to time.

Figure 9 shows changes in the permeability coefficient of the microporous ceramic filter membrane with respect to time. The variation law of the permeability coefficient as a function of time was similar to that of the permeate flux with respect to time, which decreased unevenly with the extension of time. When the seepage time reached about 100 h, the permeability coefficients of the same series of emitters under different heads were very similar. From the fitting results shown in Figure 9, the general formula of the permeability coefficient (*k*) and the overcurrent time (*t*) is easily obtained for different series of microporous ceramic filter membranes:

(7) $$k\left(t\right)=gt^{h}=\left(aL/AH\right)t^{b}.$$

**Figure 10:**
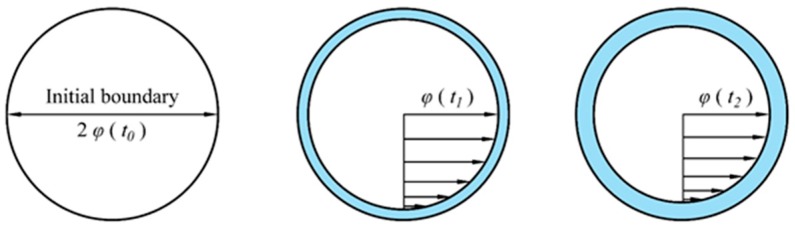
Schematic diagram of effective penetration radius change.

When seepage began, there was a small water flow along the side wall of the ceramic pores. As seepage time was prolonged, a water film gradually forms on the surface of the inner microchannel. At this point, the conditions changed along the water flow side wall, and the water flow moved relative to the water film boundary (Figure 10). To facilitate calculations, the internal water flow channel of the microporous ceramic was simplified into an equal-diameter circular tube model to analyze the hydraulic characteristics. Therefore, the loss of water flow during the flow was mainly the resistance loss along the path: *h_f_* = *λlv*^2^/2*gd* [31]. The resistance coefficient *λ* was positively correlated with roughness. It can be concluded that the resistance loss coefficient along the ceramic side wall (*λc*) was greater than the resistance loss coefficient along the side wall of the water film (*λs*) because the roughness of the ceramic side wall (∆*c*) was greater than the roughness of the water film side wall (∆*s*). When the microporous ceramic filter membrane started to seep (*t* = *t*_0_), the velocity (*v* (*t*_1_)) and the resistance loss coefficient (*λc*) along the path were large. Thus, the resistance loss (*h_f_* (*t*_1_)) was relatively large, and the permeate flux (*Q*) decreased rapidly. But the effective penetration radius (*φ* (*t*_0_)) was the largest at this time, hence the value of *Q* was large. After the initial formation of the water film (*t* = *t*_1_), *v* (*t*_1_), and *λ_s_* were relatively small, and thus, *h_f_* (*t*_1_) of water flow was relatively small, and hence, *Q* decreased slowly. At this point, *φ* (*t*_1_) was small, and thus, the value of *Q* was small. After the water film completely formed (*t* = *t*_2_), *φ* (*t*_2_), and *h_f_* (*t*_2_) tended to be stable, and hence, *Q* also tended to be stable. Therefore, there is a mathematical relationship between the change in the effective penetration radius and the change in the outflow rate of the microporous ceramic filter membrane.

The laminar flow state of the water flow inside the microporous ceramic emitter can be expressed according to Darcy’s law:

(8) Q=kAH/L

where *Q* is the flow, *k* is the permeability coefficient, *A* is the equivalent seepage area, *H* is the working pressure, and *L* is the equivalent permeability.

The relationship between the open porosity (*e*) and the effective penetration radius (*φ*) can be approximated as:

(9) $$e=\varphi^{2}/d^{2}$$

where *d* is the bottom radius of the microporous ceramic emitter.

Combining Equations (8) and (9) gives:

(10) $$k=\left(m/d^{2n}\right)\varphi^{2n}=m^{\prime }\varphi^{2n}.$$

Combining Equations (2), (3) and (10) give the relationship between the effective penetration radius (*φ*) and the time (*t*):

(11) $$at^{b}=m^{\prime }\varphi^{2n}AH/L.$$

**Figure 11:**
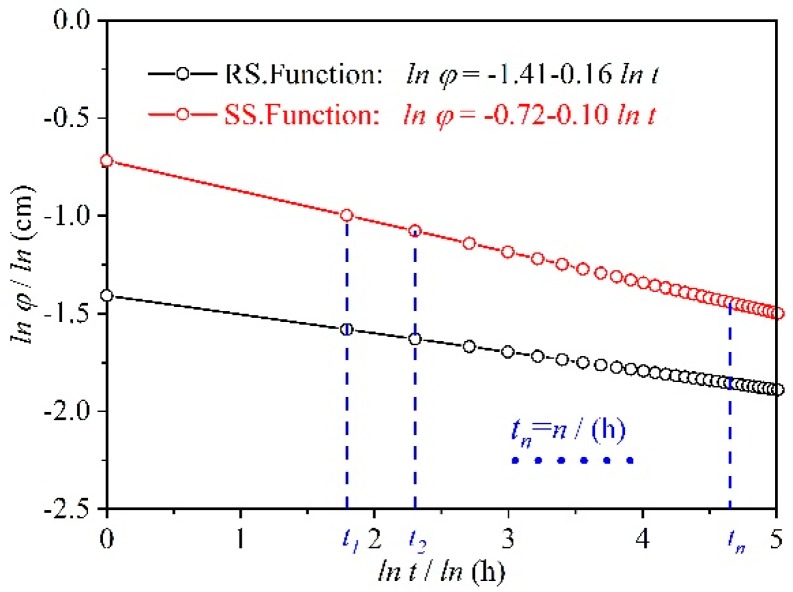
Logarithm of the effective penetration radius (*ln φ*) as a function of logarithm of the seepage time (*ln t*).

Equation (11) is converted to a logarithmic form to determine the *φ*–*t* relationship curve with as few points as possible. The logarithmic form of the Equation (11) is:

(12) $$ln\;\varphi =ln\left(aL/m^{\prime }AH\right)+bln\;t/2n=\left(P+bln\;t\right)/2n$$

where $P=ln\left(aL/m^{\prime }AH\right)$. Using the RS series and SS series microporous ceramic filter membranes as examples, the relationship curves of *ln φ*–*ln t* were plotted (Figure 11). The flow rate of the microporous ceramic could be roughly estimated from these curves. As can be seen from Figure 11, the effective penetration radius (*φ*) of the RS series was small and the range of variation with time was low. The effective penetration radius (*φ*) of the SS series was large and varieds greatly over time. Also, the effective penetration radius of RS and SS changed less and less over time.

Using Equation (12), a basic index is proposed to measure the stability of the permeate flux of microporous ceramic filter membranes:

(13) S=|aL/bAH|

where *S* < 0 and *b* < 0. When *S* is large, the difference between the value of the initial permeate flux and the value of the stable permeate flux is smaller. Over time, the uniformity of the permeate flux of the microporous ceramic filter membrane is better. When S is smaller, the difference between the initial permeate flux and the stable permeate flux is larger, and the permeation flux of the microporous ceramic filter membrane fluctuates with time. Under a working pressure of 20 cm (*S_SS_*/*S_RS_* = 0.98 < 1), the permeate flux of SS was relatively stable, and under a working pressure of 50 cm (*S_SS_*/*S_RS_* = 1.05 > 1), the permeate flux of RS was relatively stable. The two materials exhibited different penetration stabilities under different working pressures. This may occur because changes in working pressure within the flow channel cause a redistribution of the force conditions of small water flow. Combining Newton’s internal friction law (*F = μ*(*du/dy*)*A*) with Equation (9) gives: *F = μ*(*d*(*kH*)*/dy*)*A*. When the water film thickness (*dy*) does not change, the working pressure (*H*) becomes larger, the viscous force F is larger, and the water flow decreases faster. That is, the permeate flux is more unstable. With an increase in working pressure, the proportion of the viscous force increases in the overall force system of the water flow. The increase in *F_RS_* was larger than that in *F_SS_* because *μ_RS_* was greater than *μ_SS_*, and thus, *S_SS_* was greater than *S_RS_* under higher working pressure. Eventually, the penetration flux of SS was more stable.

From the above analysis process, the authors attempted to propose some approaches for improving the permeate flux of the ceramic membrane.

### 4.2. Approaches for Improving Permeate Flux

#### 4.2.1. Adjusting Porosity

As seen from Equation (7), when the porosity of the opening is larger, the initial permeate flux is larger. Additionally, the coefficient (a) is large because of the nature of the power function. According to Equation (12), the effective penetration radius is also large. The final stable permeate flux is also large. Because one of the material characteristics that is the most easily controlled in microporous ceramics is the open porosity, the preparation methods commonly used currently can easily achieve this purpose. Therefore, adjusting the porosity is one of the most convenient ways to improve permeate flux. As seen from Equations (7) and (10), as the porosity of the opening increases linearly, the overall effective penetration radius (*φ*) of the ceramic filter membrane increases, and the permeability coefficient increases exponentially. The permeability coefficient is an indication of the actual permeability of the ceramic filter membrane. Therefore, increasing the open porosity and reducing the closed porosity can effectively improve the permeate flux of the ceramic membrane.

#### 4.2.2. Improving Surface Properties of Materials

The RS series and the SS series exhibited different degrees of roughness in terms of microstructure because of the assisting effect of alkali metal ions. As seen in Figure 6f, compared with the RS series, the SS series had a complete grain structure and a clear boundary. Thus, for the SS series, the surface roughness of the side wall was relatively large, and there was an obvious blocking effect of the side wall on the water flow. Also, the loss coefficient (λ) was large. Therefore, the permeate flux (*Q*) and the effective permeation radius (*φ*) decreased at a faster rate. To reduce how fast the permeate flux and effective permeation radius decrease, it is recommended that sand with relatively complete particles be selected as the raw material. In addition, after ball milling for a long time, there is a high probability that sand and gravel are crushed. The amount of fine particles (generated by crushing) that are incorporated into the powder tends to increase the overall roughness. When the material is prepared, the surface of the sand and gravel can be manually washed to remove the finely crushed sand.

#### 4.2.3. Selecting an Appropriate Matrix Material and Pore Former

The hydrophilicity of the material affects the size of the stable effective penetration radius. Hydrophilic materials with polar group molecules have a greater affinity for water. Water forms a contact angle of more than 90° on the surface of a hydrophobic material, and therefore, the surface of a hydrophilic material is more likely to form a water film. The microporous ceramic filter membrane prepared using the hydrophilic material has a larger variation in the effective penetration radius because of temporary hydrogen bonding between water and the polar groups of the material, and this means that the permeate flux occurs over time. When the magnitude of change is greater, the effective penetration radius that ultimately forms is smaller. For example, after high-temperature sintering, the RS series of fused silica forms a large amount of glass phase after cooling, and glass is a typical hydrophilic material. Therefore, compared with the SS series, the RS series had a small effective penetration radius (*φ*) (Figure 11). Microporous ceramics are mainly composed of a crystalline phase, glass phase, and pores, and the hydrophilicity is determined by the ratio of the crystalline phase to the glass phase. When the amount of alkali metal ions in the matrix material is high, the crystal melting temperature is low, and the glass phase forms easily (Figure 6b). Conversely, when the amount of impurities is low, the crystal structure of the quartz particles is relatively intact at high temperature. It is not easily destroyed (Figure 6f). In summary, it is recommended that sand with higher purity should be selected as a matrix material, and that an organic pore-forming agent (such as sodium carboxymethyl cellulose) should be selected as the type of pore-forming agent.

## 5. Conclusions

In the paper, the relationship between hydraulic performance and the material properties of the microporous ceramic filtration membranes was studied. Glass phase in microporous ceramics led to an increase in the viscous drag effect of the side wall on the water flow, and the wall roughness inside the pore increased the resistance of the flow of fine water. They led to a layer of water film to adhere to the boundary inside the pore. Based on this, a semi-empirical formula was constructed and expresses the effective permeation radius as a function of seepage time: *ln φ* = (*P* + *bln t*)/2*n*. A basic index was proposed to measure the permeate flux stability of microporous ceramic filter membranes: *S* = |*aL*/*bAH*|. Then, on the basis of theoretical analysis, some approaches for improving the permeate flux were proposed: (1) adjusting porosity, (2) improving surface properties of materials, and (3) selecting an appropriate matrix material and pore former. Finally, the formation of the water film in the inner wall of the pore is a very complicated and interesting process. In future research, some work can be done in related areas.

## Figures and Tables

**Table 1:** Chemical composition of raw materials.

Raw Material	Chemical Composition							
	*ω*(SiO_2_) %	*ω*(Al_2_O_3_) %	*ω*(Fe_2_O_3_) %	*ω*(CaO) %	*ω*(MgO) %	*ω*(K_2_O) %	*ω*(Na_2_O) %	Others
River sand	90.34	1.92	0.37	2.33	0.24	0.93	0.82	3.29
Standard sand	98.88	-	-	-	-	-	-	1.12

**Table 2:** Formulation of the samples.

Samples	*ω*(Standard Sand) %	*ω*(River Sand) %	*ω*(Alumina) %	*ω*(Slag) %	*ω*(Talcum) %	Total
RS	-	63.17	7.46	5.83	25.54	100
SS	63.17	-	7.46	5.83	25.54	100

***ω***—Quality score.
